# Gold nanoparticle-mediated non-covalent functionalization of graphene for field-effect transistors†

**DOI:** 10.1039/d0na00603c

**Published:** 2021-01-08

**Authors:** Dongha Shin, Hwa Rang Kim, Byung Hee Hong

**Affiliations:** Division of Fine Chemistry and Engineering, Pai Chai University Daejeon 35345 Republic of Korea; Department of Chemistry, Seoul National University Seoul 08826 Korea; Graphene Research Center & Graphene Square Inc., Advanced Institute of Convergence Technology, Seoul National University Suwon 16229 Korea

## Abstract

Since its discovery, graphene has attracted much attention due to its unique electrical transport properties that can be applied to high-performance field-effect transistors (FETs). However, mounting chemical functionalities onto graphene inevitably involves the breaking of sp^2^ bonds, resulting in the degradation of the mechanical and electrical properties compared to pristine graphene. Here, we report a new strategy to chemically functionalize graphene for use in FETs without affecting the electrical performance. The key idea is to control the Fermi level of the graphene using the consecutive treatment of gold nanoparticles (AuNPs) and thiol-SAM (self-assembled monolayer) molecules, inducing positive and negative doping effects, respectively, by flipping the electric dipoles between AuNPs and SAMs. Based on this method, we demonstrate a Dirac voltage switcher on a graphene FET using heavy metal ions on functionalized graphene, where the carboxyl functional groups of the mediating SAMs efficiently form complexes with the metal ions and, as a result, the Dirac voltage can be positively shifted by different charge doping on graphene. We believe that the nanoparticle-mediated SAM functionalization of graphene can pave the way to developing high-performance chemical, environmental, and biological sensors that fully utilize the pristine properties of graphene.

## Abstract 2

Consecutive treatment of gold nanoparticles and thiol-SAM molecules induce the recovery of electronic state of pristine graphene.

## Graphical abstract

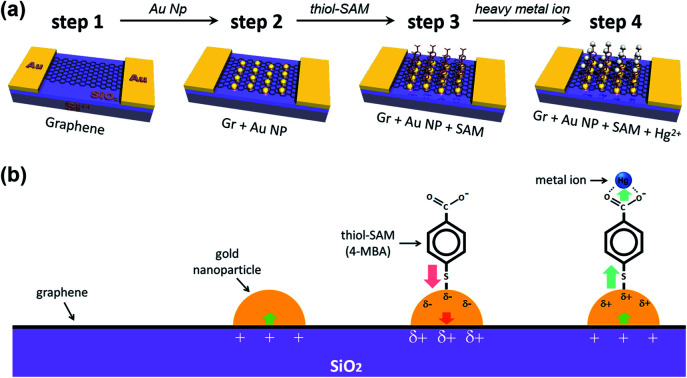

Graphene was the first of the recently realized ideal two dimensional materials.^1^ Its extraordinary mechanical,^2^ electrical^3–5^ and optical properties^6–8^ have led to a variety of novel new devices, such as flexible transistors,^9^ ultrafast lasers,^10^ photodetectors^11,12^ and optical modulators.^13,14^ Among these, the ultrahigh carrier mobility (both electron and hole) of graphene makes it a promising material for nanoelectronic devices.^5,15,16^ The upper limit of the outstanding electrical properties of a graphene-based field effect transistor (FET) is already well-known.^17,18^ Much effort has been done to develop new graphene FETs for suitable purposes. Chemical functionalization can allow graphene FETs to be realized as various chemical^19–31^ and biological sensing^32–41^ platforms. The most frequent way to implement such functions has been accomplished by the direct covalent bonding of molecules to graphene,^19,20,42–46^ such as through an azide group.^47,48^

This method, however, converts the sp^2^ hybrid orbital, which is the intrinsic characteristic of graphene, to a broken sp^3^ character, and thus inevitably degrades the pristine electrical property of graphene.^42^ In addition, if not that severe, adopting the heterogeneous species including functional molecules, in general, cannot avoid a significant doping effect, which substantially shifts the charge neutrality point (*V*_CNP_) (Dirac point voltage) from the pristine value and limits the detection range in real applications. Thus, our interest is to determine how to introduce functional groups on graphene FETs without impairing the pristine sp^2^ orbitals.

In this work, we have proposed a novel method to modulate the electrical properties of graphene FET devices by adopting gold nanoparticles and thiol-SAM molecules. Consecutive treatment of the nanoparticle and SAM molecules induces the p-type and n-type doping effects, respectively. By analyzing the doped characteristics both electrically and optically, we realized a functionalized graphene FET device that preserves the close-pristine electronic state of graphene.

Fig. 1 schematically shows the process of sequential treatment on a graphene surface. Firstly, we prepared patterned gold electrodes on silicon oxide (300 nm)/silicon (p-type) (SiO_2_/Si) wafers, and then the as-prepared CVD (chemical vapor deposition) graphene was transferred onto Au electrodes (step 1). Next, we incorporated the gold nanoparticles onto the graphene surface, and this is the key step (step 2) in our experiments. As shown by the step 3 process in Fig. 1, the gold nanoparticles were deposited onto the graphene surface by the spontaneous reduction of a solution-based metal precursor (AuCl_4_^−^), induced by the redox potential difference (the galvanic exchange) between them.^49,50^ This electroless method is cost-effective and straightforward since it does not require any extra linking molecules or reducing agents. Only the dipping time, along with precursor concentration (in stock solution), needed to be controlled (Fig. S1†). For the same dipping time, it was found that the higher the concentration of HAuCl_4_, the higher the shift of *V*_CNP_, and the devices were destroyed by the high voltage near a concentration of 1 mM, making it impossible to measure *V*_CNP_. Thus, several attempts were made to determine the appropriate concentration of the precursor of the AuNPs. More detailed studies on such spontaneous reductions of gold nanoparticles, such as substrate dependency, have been reported in previous papers.^49,50^ AFM (atomic force microscopy) analysis confirmed that our deposited gold nanoparticles are uniformly distributed, at ∼4 nm in height (Fig. S2†).

**Fig. 1:**
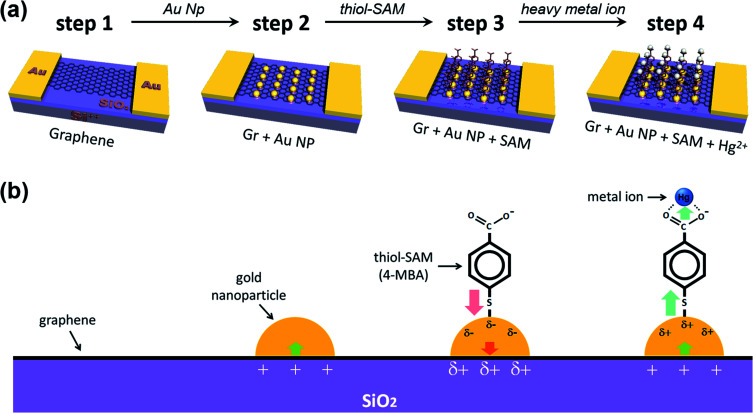
(a) Schematic diagram to show the fabrication process of a graphene FET device and (b) enlarged views, denoting the configurations and charge transfer direction in each step. Deposited gold nanoparticles induce the partial p-type doping on graphene (step 2), while the thiol-SAM molecules (4-mercapto benzoic acid, 4-MBA) induce n-type doping (step 3). Step 4 shows that the captured heavy metal ion induces p-type doping on graphene (step 4).

X-ray photoelectron spectroscopy (XPS) was used to verify that the desired treatment of each step was achieved through the dipping method. The spectra on the far left of each row in Fig. 2 represent the wide scan range of XPS at each step (Fig. 2a, c, f and j). Graphitic carbon sp^2^ bonding in monolayer graphene represents an asymmetric curve in the XPS spectra due to intrinsic interactions between the inner core hole and the electrons in the valence band.^51–54^Fig. 2b reflects this and indicates that the pristine graphene by the CVD method is well-formed without breaking its hexagonal symmetry. In addition, Fig. 2d, g and k show that the symmetry is not broken by the nanocomponent treated at each stage and remains intact.

**Fig. 2:**
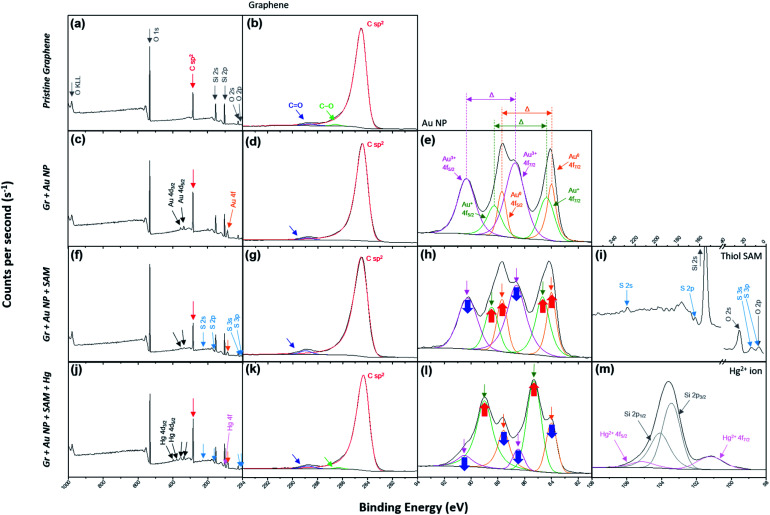
(a) The wide scan and (b) C 1s range for the XPS of the pristine graphene film (step 1 in Fig. 1). (c) The wide scan, (d) C 1s and (e) Au 4f ranges for the XPS of Gr + Au (step 2 in Fig. 1). (f) The wide scan, (g) C 1s, (h) Au 4f and (i) S 2p ranges for the XPS of Gr + Au + SAM (step 3 in Fig. 1). (j) The wide scan, (k) C 1s, (l) Au 4f and (m) Hg 4f ranges for the XPS of Gr + Au + SAM + Hg (step 4 in Fig. 1).

The process of forming gold nanoparticles above graphene is extremely interesting, and the XPS spectra results of the Au 4f levels show that three chemical species of gold exist. From the results in Fig. 2e, we can see that the Au ions that disassembled from HAuCl_4_ exist in the Au^3+^ state, and when they are reduced to Au NPs, they exist in the Au^0^ state, or some in the Au^+^ state. Each binding energy corresponding to 4f_7/2_ of Au^0^, Au^+^, and Au^3+^ is about 84–84.5, 85–86, and 86–87 eV.^55–59^ In Fig. 2e (as well as in Fig. 2h and l), the energy difference, *Δ*, is separated in the Au 4f region due to orbital energy splitting as a result of spin–orbit coupling in the 4f orbital of the heavy metal elements. The value of *Δ* between Au 4f_7/2_ and 4f_5/2_ is 3.67 eV,^55,57^ and this can be found from XPS spectra for the Au NPs. In this process, Au NPs are reduced by the graphene film and the surface of graphene is doped as p-type (step 2 in Fig. 1).

In addition, sulfur peaks of thiol SAM which were not found in the wide scan range of the XPS spectra in the previous process, were found as weak signals in Fig. 2f and i. Those values correspond exactly to the S 2p level between a binding energy of 161 and 164 eV.^60^ In Fig. 2h, it can be observed that the intensity of the Au^3+^ peak has decreased, while the intensities of the Au^0^ and Au^+^ peaks have increased, due to the negative charge of the carboxyl group in SAM (step 3 in Fig. 1).

In order to investigate the electrical properties at each step, we conducted FET measurements using a three-point probe station at ambient conditions. Fig. 3a shows that the representative electrical transfer curve (characteristics) in pristine graphene (black, Gr) has shifted to the right, representing p-type doping (blue arrow, ①) of graphene (orange curve, Gr + Au).

**Fig. 3:**
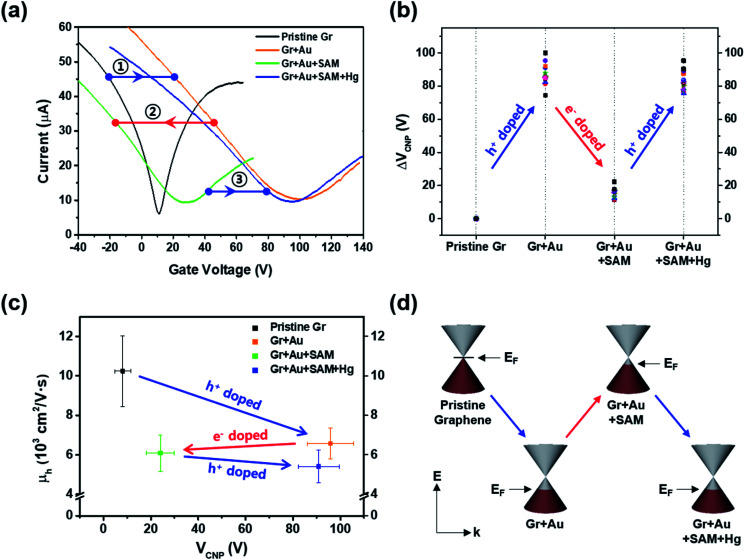
(a) Representative electrical transfer curves of graphene FET devices measured at each doping step. (b) Charge neutrality point (*V*_CNP_) variation at each step. (c) Hole carrier mobility (*μ*_h_) plot with respect to *V*_CNP_. (d) Schematic illustration of the band structures and the variation in the Fermi energy levels (EF) of graphene.

For the next step, we carried out molecular functionalization onto this substrate (step 3 in Fig. 1). Rather than using conventional azide group-containing molecules, we chose more convenient thiol molecules that can form a spontaneously self-assembled monolayer on the gold surface through Au–S bonds. Due to its self-assembling character, the molecule should have a vertical (slightly tilted) standing configuration, and without the gold surface this molecule should be deposited onto the graphene surface directly *via* π–π interactions, favoring a horizontal laying-down configuration. As a control experiment, we tested the effect of thiol-SAM treatment on graphene without a gold nanoparticle deposition step, and this revealed that thiol-SAM induces an opposite hole doping effect on graphene (see Fig. S3a†). Interestingly, we can observe from Fig. 3a that thiol-SAM treatment has moved the transfer curve of the gold nanoparticle-adsorbed state (orange, Gr + Au) to the left, and this means the induction of the electron doping effect (red arrow, ②) on graphene (green curve, Gr + Au + SAM). According to previous reports,^61–64^ noble metal nanoparticles are highly susceptible to interactions with external molecules, resulting in a surface potential (electronic state) change depending on the character of the adsorbed molecule. It has been known that thiol functionalization is likely to induce a negative doping effect, which is electron donating, on the gold nanoparticle surface.^65^ Such SAM-induced electron donation on the gold nanoparticle then concurrently induces negatively charged doping (n-type doping) on the graphene. Therefore, such succeeding p-type (Au nanoparticles) and n-type (thiol-SAM) doping effects finally result in the restoration of the electronic state of graphene that is close to its pristine state, and this can be used as a metal ion captor in the next step through the functionalized pendent chemical group. In order to confirm its generality, we also compared the FET responses using other kinds of thiol-SAM molecules, showing that all of the thiol-SAM molecules induce a similar n-type doping effect on graphene, while exhibiting different doping degrees depending on the molecular type. Such differences might be associated with molecular dipole moments^65^ (Fig. S4†).

Next, we demonstrated the performance of our graphene FET [pendent carboxyl group of 4-mercapto benzoic acid (4-MBA)] as a mercury ion captor. Step 4 in Fig. 1 shows the capture of a mercury ion by the carboxyl group in our FET platform. It is well-known that carboxyl groups make complexes (chelating bidentate forms) with various transition metal cations due to the high stability constant of the reactions.^66–68^ Hg^2+^ ions act as bidentate ligands in this step. In addition, the XPS results show that the Hg 4f region is separated by 4.05 eV energy splitting, the same as the Au 4f region.^69^ In addition, since the substrates used in the study were SiO_2_/Si substrates, we must be careful about overlapping Si 2p peak positions in the XPS analysis of Hg 4f.^70,71^

Thus, after dipping the substrate (step 3 in Fig. 1) into the mercury ion solution, the measured FET character showed a shift (blue arrow, ③) of the transfer curve to the p-type doped state (right side) of graphene (blue curve in Fig. 3a). This can be seen by the further decrease in the intensity of the Au^0^ peak in Fig. 2l, and that the intensity of the Au^+^ peak increased significantly. As a control experiment, we confirmed that without 4-MBA SAM treatment, a mercury ion solely induces an opposite negative (electron) doping effect on gold nanoparticle-deposited graphene FETs (see Fig. S3b†). In addition, we also observed that 4-mercaptotoluene (4-MT)-based FETs show a negligible response to mercury ion treatment, verifying the strong interaction between the carboxyl group of 4-MBA and the mercury ion (Fig. S5†).

Fig. 3b shows the change in charge neutrality point (Δ*V*_CNP_) value at each step. Although some variation was observed (device to device variation) at each step, sequential treatments generally induced the change in doping state in graphene, from pristine to p-type doped to n-type doped to p-type doped (*V*_CNP_: 8.14 → 95.86 → 24.03 → 90.86 V). We calculated the hole mobility of the doped graphene, estimated using the standard model of the metal-oxide–semiconductor FET (MOSFET). The corresponding band structures at the *K* point in the Brillouin zone of the pristine monolayer graphene are shown in Fig. 3d.

In the linear region of the transfer curve (also known as the ohmic mode), the current from drain to source, *I*_DS_, is calculated using the approximation of:
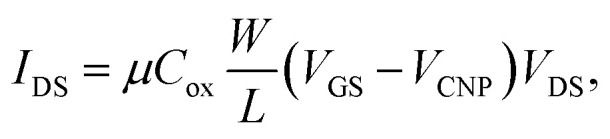
where *μ* is the carrier mobility, *C*_ox_ is the capacitance of the gate material, 300 nm of SiO_2_ (1.08 × 10^−8^ F cm^−2^), *W* is the gate width (100 μm), *L* is the gate length (40 μm), *V*_GS_ is the applied gate voltage and *V*_DS_ is the drain-source voltage difference (1 mV). The carrier mobility finally becomes:
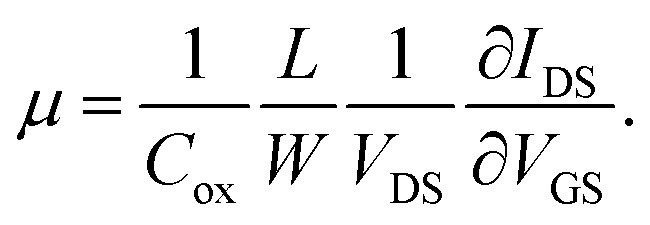


Using this equation, the hole carrier mobility, *μ*_h_, of graphene can be measured indirectly. Fig. 3c shows the mobility of each step with corresponding *V*_CNP_ values: 10 236 → 6563.1 → 6087.3 → 5401.2 cm^2^ V^−1^ s^−1^. Compared with a number of previous reports,^5,72,73^ our system shows relatively high mobility, which is advantageous in sensing capability. This can be attributed to the low charge puddles or surface defect concentration (see Fig. 4d for the low *I*_D_/*I*_G_ ratio) induced by the unbroken sp^2^ bond character.

**Fig. 4:**
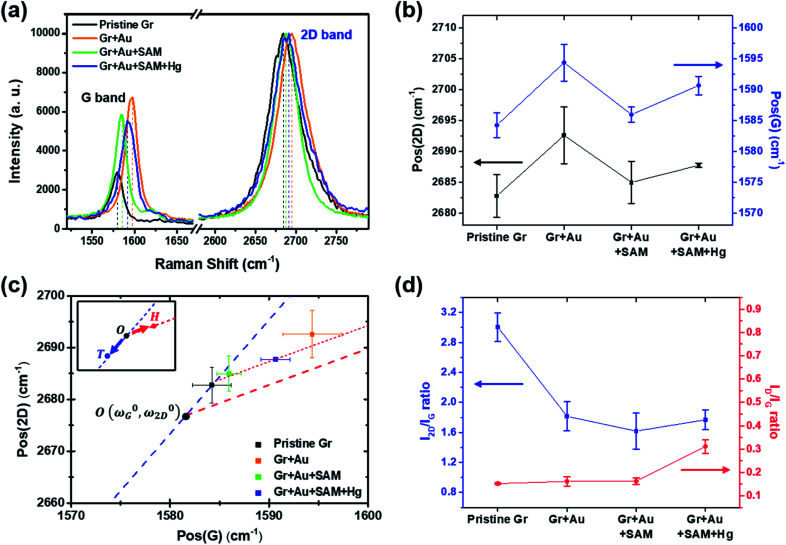
(a) Measured Raman signals for graphene FET devices at each doping step. Only the G and 2D peaks are shown for comparison. (b) Peak positional changes of the G and 2D peaks at each step. (c) The 2D and G peak position plot, revealing that our treatment induces charge doping rather than a strain effect (from ref. 40). (d) The variation in the *I*_2D_/*I*_G_ and *I*_D_/*I*_G_ ratios at each step.

Raman spectroscopy is a valuable optical technique for analyzing the electrical properties of graphene.^74^Fig. 4 shows the Raman analyses of graphene FET devices at each step, measured at ambient conditions. For all measurements, we used 514.5 nm laser light (<1 mW) with a spot size of 2 μm to reduce the damage on the samples. First of all, it should be noted that the original (not in ours) pristine graphene that is strain-free and charge-neutral shows the G and 2D bands at 1581.6 ± 0.2 and 2676.9 ± 0.7 cm^−1^, respectively,^75^ and such G and 2D peaks signify the frequencies of the phonon interactions at the *Γ* and *K* points in the Brillouin zone, respectively. In our case, as shown in Fig. 4a and b, the pristine graphene shows G and 2D peak positions at 1584.21 ± 1.98 cm^−1^ and 2682.75 ± 3.47 cm^−1^, respectively. After the gold nanoparticles were deposited, the peaks shifted to the higher positions at 1594.36 ± 2.98 cm^−1^ and 2692.59 ± 4.59 cm^−1^ and, after thiol-SAM (4-MBA) treatment, returned to 1585.95 ± 1.22 and 2684.96 ± 2.98 cm^−1^, respectively. Finally, mercury ion treatment induces a shift of the G and 2D peak positions to 1590.64 ± 1.50 and 2687.72 ± 0.34 cm^−1^, respectively.

According to Lee *et al.*,^75^ Raman spectroscopy can be used to optically differentiate between the strain effect and the charge doping effect in graphene samples. Fig. 4c shows the G *versus* 2D peak position of our samples, indicating that the point of *O*(*ω*^0^_G_,*ω*^0^_2D_) (1581.6 ± 0.2, 2676.9 ± 0.7) originally corresponds to the strain-free and charge-neutral state. If tensile strain or a p-type doping effect is applied to this graphene, the point (*ω*_G_,*ω*_2D_) moves in the direction 
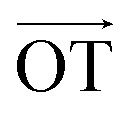
 or 
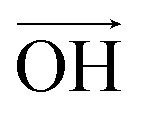
, respectively (inset of Fig. 4c, where the slopes are 2.2 ± 0.2 and 0.70 ± 0.05 for 
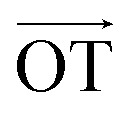
 and 
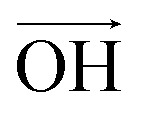
, respectively^75^). Even though our pristine graphene state (black square) is slightly far away from the original point of *O*(*ω*^0^_G_,*ω*^0^_2D_), it is still positioned along the blue dashed line, which means that only compressive strain has been applied to our pristine sample. Since graphene has an intrinsically negative thermal expansion coefficient in the range of 200–400 °C,^76–79^ annealing at 300 °C and cooling to room temperature unavoidably results in compressive strain on graphene, and this is the situation in our case. Next, our experiments showed that, in the consecutive treatment of the gold nanoparticles, thiol-SAM molecules and mercury ions, the graphene states in the plot (Fig. 4c) change from black to orange, green, and finally to blue squares. It should be noted that all these states are still along the red dashed line, indicating that all the treatments only result in a charge-induced doping effect on graphene, and not a mechanical strain effect. These results correlate well with the shifts of *V*_CNP_ in graphene FETs.

On the other hand, the intensity ratio between the 2D and G peaks (*I*_2D_/*I*_G_) is also a good parameter to evaluate the doping strength in a graphene sample. Fig. 4d shows that it decreased from 3.004 ± 0.193 for the pristine state (step 1) to 1.769 ± 0.132 for the mercury ion-treated state (step 4). Furthermore, it is well-known that the D band in Raman spectra is related to the defect density of graphene films. In all of our treatments, the intensity ratio of the D to G peak has remained relatively small in the range of 0.152 ± 0.004 to 0.311 ± 0.029, indicating that only small defects are generated on graphene.

In the Raman analyses of Fig. 4, we only used a laser with a wavelength of 514 nm. However, in order to observe the surface-enhanced Raman scattering (SERS) signal by gold nanoparticles,^80^ we should use a 633 nm laser instead of a 514 nm laser. In Fig. S7,† the Raman spectra with a full range (100 to 3200 cm^−1^) of pristine graphene is compared with those with the 514 nm and 633 nm lasers. In the upper figure (514 nm), we can clearly observe the G and 2D peak characteristics of graphene, but in the lower figure (633 nm), the intensities of the two peaks are relatively low. Therefore, we used the 514 nm laser in this research. To observe the SERS effect by the Au NPs, the Raman spectra of each step (in Fig. 1) with a 633 nm laser are shown in Fig. S8.†^49,50,64,81,82^ At a Raman shift between 1000 and 1700 cm^−1^, we can observe the Raman peak of the Au NPs, which was invisible in the case of pristine graphene. When thiol SAM is combined^62,63,83–85^ or a Hg^2+^ ion is chelate,^86–90^ a much stronger peak intensity was observed by the surface-enhanced effect of the Au NPs. Furthermore, the SERS effect by thiol chemisorption on Au NPs was observed at a Raman shift of 175–525 cm^−1^, as shown in Fig. S8c and d.†^63^ In Fig. S7a† (514 nm), the positions of the red arrows are the intrinsic peaks of the silicon nanostructure (substrate).^91^ It is necessary to pay attention to Fig. S83 and S84.† When adding the Hg^2+^ ions, they are chelated by carboxyl groups of thiol SAM molecules bound to the Au NPs. The symmetric stretching mode of COO^−^ is blue shifted, which results in the formation of a chelating (bidentate) structure rather than a unidentate or bridging structure.^83^ Moreover, in the same process, the peak near 1630 cm^−1^ was removed because the stretching mode of C=O in the carboxyl acid form disappeared as it formed the bidentate form.^83,88^

Fig. 5 compares the charge carrier concentration of the graphene sample, which is estimated from the shifts of the *V*_CNP_ and G peak positions.^92–94^ In the standard carrier density model,^94,95^ the hole concentration of the graphene film is quantitatively calculated by the following equation:
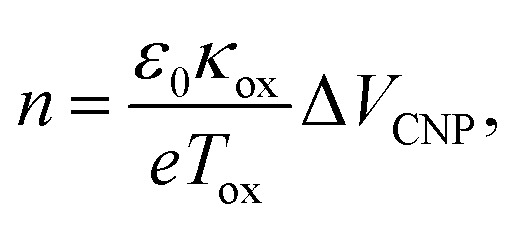
where *ε*_0_ is the vacuum permittivity (8.854 × 10^−12^ F m^−1^), *κ*_ox_ is the relative permittivity of the gate material, 300 nm of SiO_2_ (3.9), *e* is the elementary charge (1.602 × 10^−19^ C) and *T*_ox_ is the thickness of the gate material. In this experiment, the gate material was SiO_2_ and its thickness, *T*_ox_, was 300 nm. Generally, in Raman spectra of graphene, the G peak can be used to measure the charge concentration,^93^ indicating that 1 × 10^12^ cm^−2^ of the hole concentration corresponds to 1–3 cm^−1^ of the G peak positional measurements (1 cm^−1^). Therefore, we can demonstrate that the carrier concentrations, *n*, derived by Raman measurements, are comparable to the value for FET, showing the reliability of our FET measurements in each step.

**Fig. 5:**
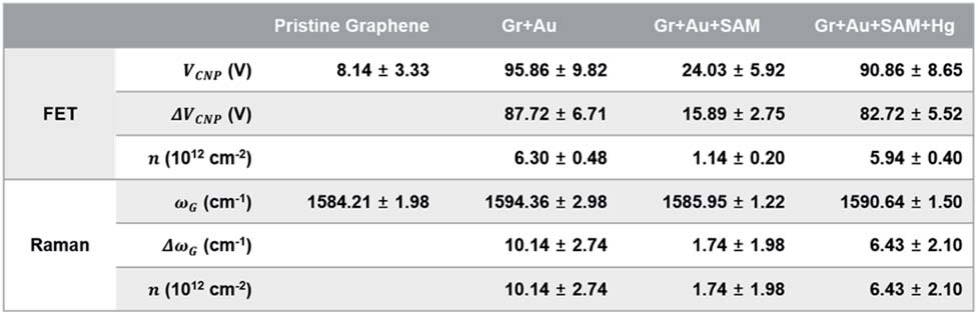
Estimated hole concentrations (*n*) at each step, derived from the shift values of *V*_CNP_ and the G peak positions.

We performed Raman spectral mapping, using a 514 nm laser, on graphene films throughout each step in Fig. 1. Raman spectral mapping images in Fig. 6a show a summary of the whole results of Fig. 4. From this, we have optically confirmed that the graphene films have clearly been doped by each nanocomponent.

**Fig. 6:**
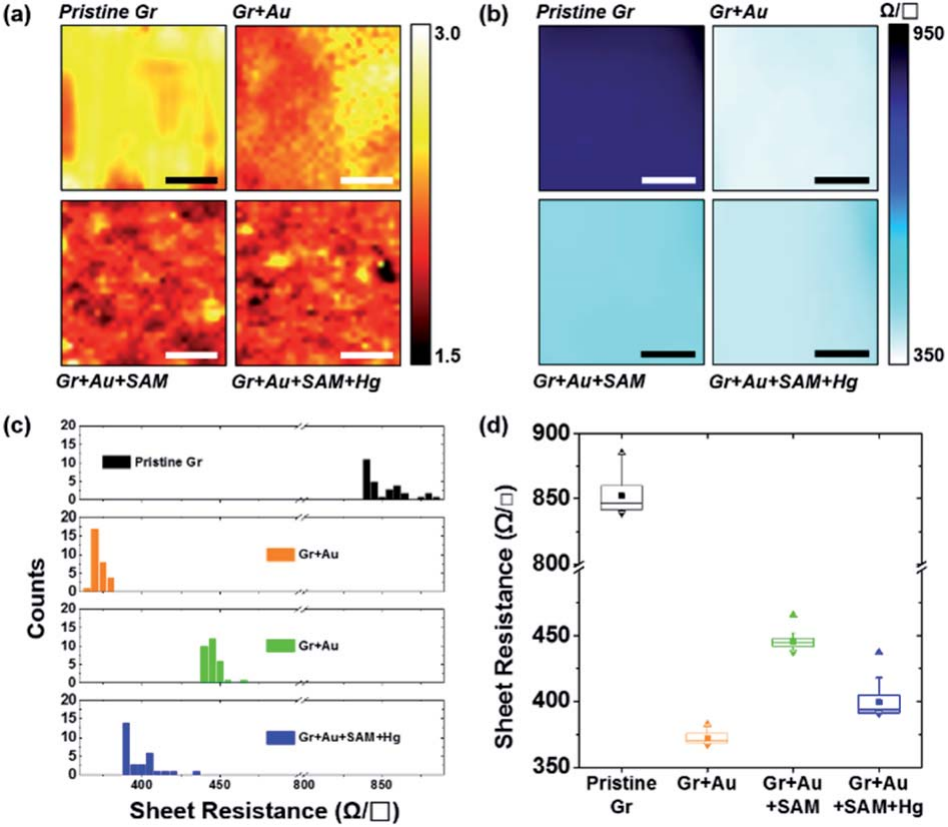
(a) Raman spectral mapping images (scale bar is 5 μm) of graphene film on SiO_2_/Si substrates for each step in Fig. 1. (b) Sheet resistance mapping images (scale bar is 1 cm), (c) a histogram of the sheet resistance, and (d) the plots for the averages and distributions of the sheet resistance of graphene film on PET substrates for each step in Fig. 1.

Fig. 6b represents the sheet resistance (*R*_S_) of graphene films, as another form of mapping image. A square graphene film with one side of 5 cm was transferred onto the PET substrate, and after doping by each step process (as shown in Fig. 1), the sheet resistance of the center area (see the yellow square in Fig. S6†) with one side of 3 cm was measured using a non-destructive method that applies magnetic fields to generate eddy current. In the case of pristine graphene, *R*_S_ is 849.50 ± 13.25 Ω □^−1^. The other cases however, in turn, are 372.85 ± 5.09, 445.53 ± 6.31, and 400.68 ± 16.93 Ω □^−1^, respectively. These results, from 30 different positions of the graphene films in Fig. S6,† of measurements using the 4-point probe method, depict the same patterns as the mapping. Fig. 6c and d show the *R*_S_ values of each step: 849.50 ± 13.43 → 372.01 ± 4.19 → 445.44 ± 5.97 → 399.11 ± 10.79 Ω □^−1^. These can be said to have the same values within a margin of error.

From Fig. 6d in particular, it can be observed that change patterns of the sheet resistance values are similar to the change patterns of the hole carrier mobility in Fig. 3c, and the *I*_2D_/*I*_G_ values of the Raman spectra in Fig. 4d. This suggests that each property of the graphene surface that is affected by doping for each nanocomponent is correlated with each other.

As already mentioned above (see Fig. S5†), in our FET device, exchanging the metal specific-carboxyl group with a non-specific methyl group substantially decreases the response of *V*_CNP_ to the mercury ion, verifying the strong interaction between the carboxyl group with the mercury ion. Then, as a next step, we conducted experiments to compare the effects of different types of heavy metal ions on the carboxyl functional group. Fig. 7 shows the different doping degrees depending on the metal type, whilst all still showing the substantial hole doping effect and highly correlated behavior between *V*_CNP_ and the Raman peak position.

**Fig. 7:**
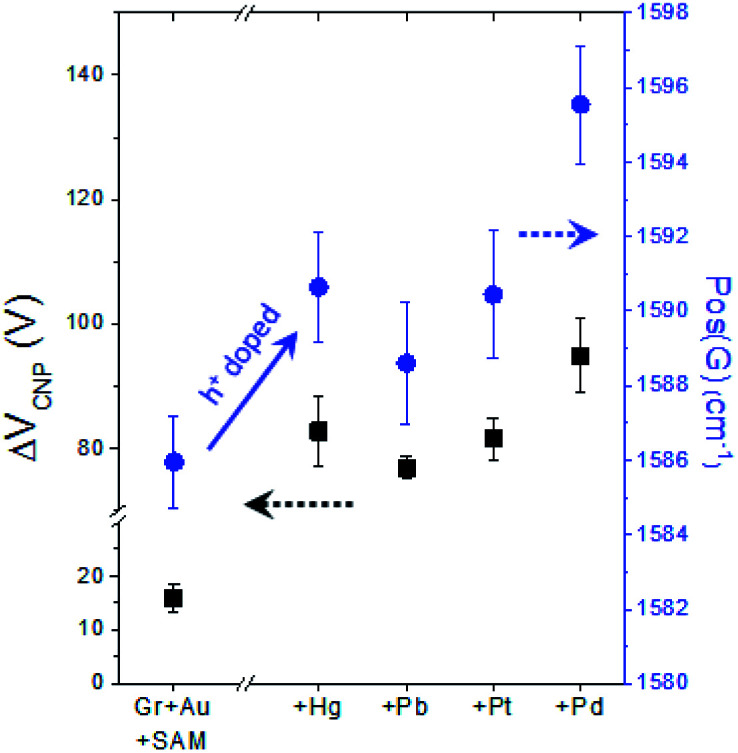
The four kinds of heavy metal ions showing a substantial hole doping effect on graphene (through the 4-MBA and gold nanoparticles) and exhibiting the highly correlated behavior of Δ*V*_CNP_ and Raman peak positions with respect to the metal types.

## Conclusions

In summary, we have developed a novel strategy to implement chemical functionality on graphene FETs with the fine tuning of doping effects on graphene. Compared to the conventional destructive direct covalent bond formation (through azide groups) on the basal plane of graphene, AuNP-mediated thiol-SAM functionalization maintains the mechanical and electrical properties of pristine graphene without affecting the sp^2^ characteristics of hexagonal carbon lattices, allowing for its application as a high-performance Dirac voltage switcher for FETs. The analyses of Raman spectra confirm that the AuNP–SAM functionalization induces a clear charge doping effect on graphene without the formation of defects, and the estimated charge carrier concentration matches well with the one from electrical transport measurements in the FETs. These results were also consistent with changes in the values of the sheet resistance. Considering the variety of chemical functional groups in SAMs that can be combined to AuNPs, our strategy is expected to provide a new route to develop highly potent graphene FETs in the near future.

## Conflicts of interest

There are no conflicts to declare.

## Supplementary Material

NA-003-D0NA00603C-s001
