# Supplementary material for: Gold nanoparticle-mediated non-covalent functionalization of graphene for field-effect transistors
Source: Nanoscale Adv. 2021 Jan 8;3(5):1404–12. doi: 10.1039/d0na00603c (PMC9419278; doi:10.1039/d0na00603c)
Supplement: NA-003-D0NA00603C-s001 [file NA-003-D0NA00603C-s001.pdf]

Electronic Supplementary Material (ESI) for Nanoscale Advances.
This journal is © The Royal Society of Chemistry 2021

Supporting Information

Gold nanoparticle-mediated chemical functionalization on graphene field effect transistor

*Dongha Shin,[‡a] Hwa Rang Kim[‡b,c] and Byung Hee Hong[*b,c]*

[‡]*These authors contribute equally*

[a]*Division of Fine Chemistry and Engineering, Pai Chai University, Daejeon 35345, Korea.*

[b]*Department of Chemistry, Seoul National University, Seoul 08826, Korea.*

[c]*Graphene Research Center & Graphene Square Inc., Advanced Institute of Convergence Technology, Seoul National University, Suwon 16229, Korea.*

[*]Correspondence to Byung Hee Hong (byunghee@snu.ac.kr)

Table of Contents

Fig. S1

Fig. S2

Fig. S3

Fig. S4

Fig. S5

Fig. S6

Fig. S7

Fig. S8

Note S1 Synthesis of graphene

Note S2 Transfer and fabrication of graphene FET devices

Note S3 Doping of graphene

Note S4 Characterization of graphene films

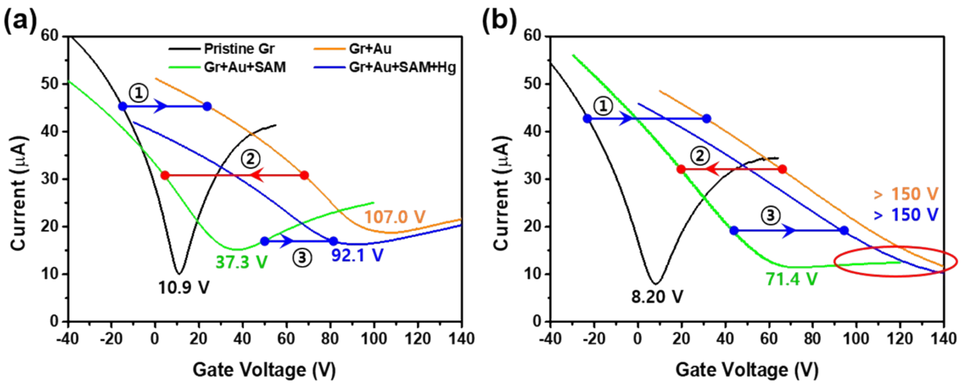

Fig. S1 The more concentrated chloroauric acid ($HAuCl_4$) (aq) induces more hole (p) doping effect on graphene FET device. (a) 0.1 mM (original conditions) and (b) 1 mM. (compare the shift ① in blue arrow)

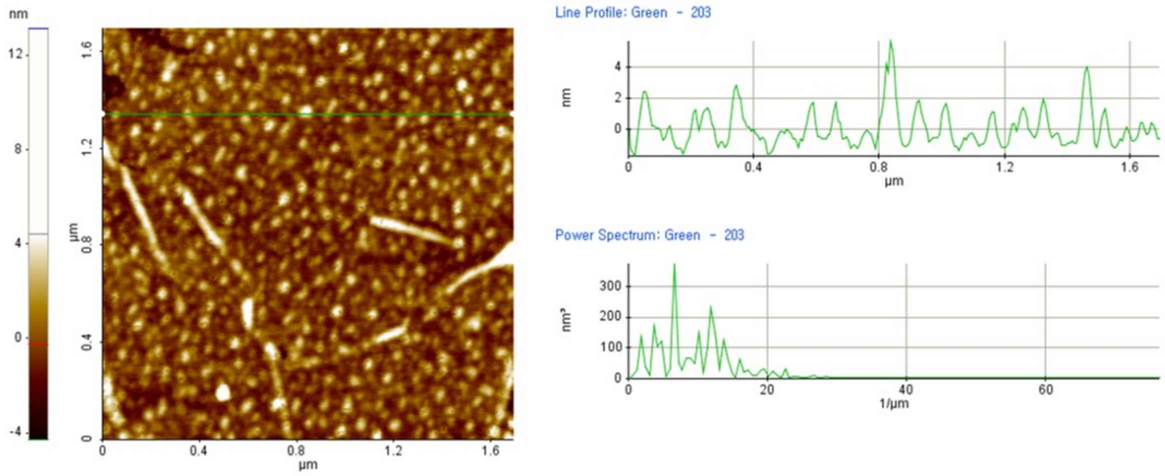

Fig. S2 AFM (atomic force microscopy) analysis shows that deposited gold nanoparticles are uniformly distributed with having ~4 nm height.

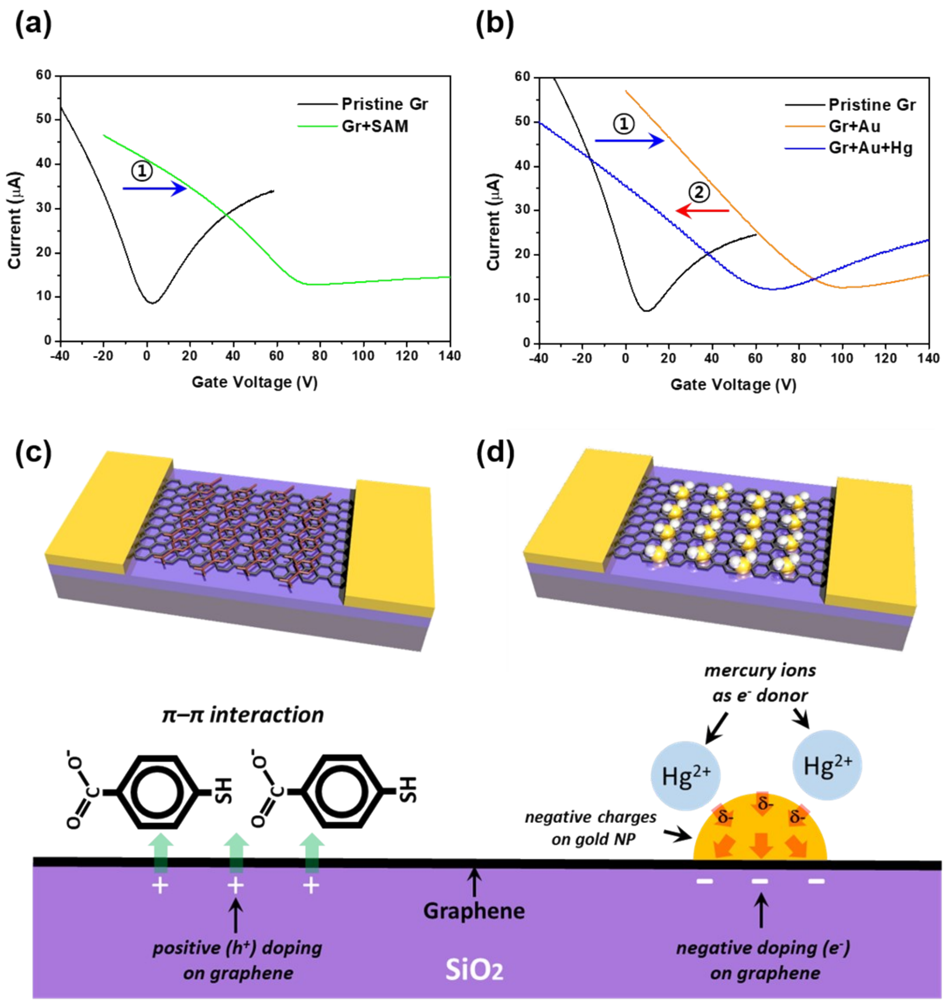

Fig. S3 The electrical characteristics of graphene FET devices, (a) treated by only 4-MBA molecule directly on graphene and (b) treated by mercury ion on gold nanoparticle without 4-MBA SAM. (c) and (d) schematically show the corresponding situation of (a) and (b), respectively.

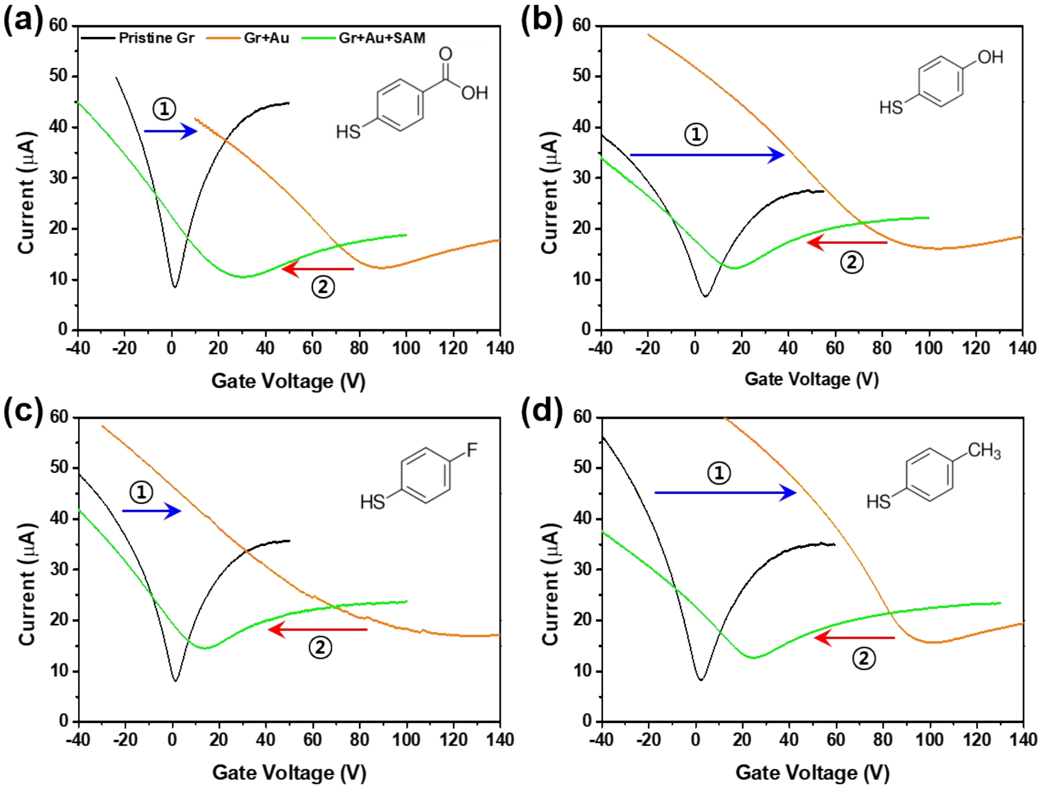

Fig S4. The electrical transfer curves show that the various thiol-SAM molecules induce universal negative doping effect on graphene through the pre-deposited gold nanoparticle. (a) 4-mercaptobenzoic acid, (b) 4-mercaptophenol, (c) 4-mercaptofluorobenzene and (d) 4-mercaptotoluene. All other experimental conditions are the same.

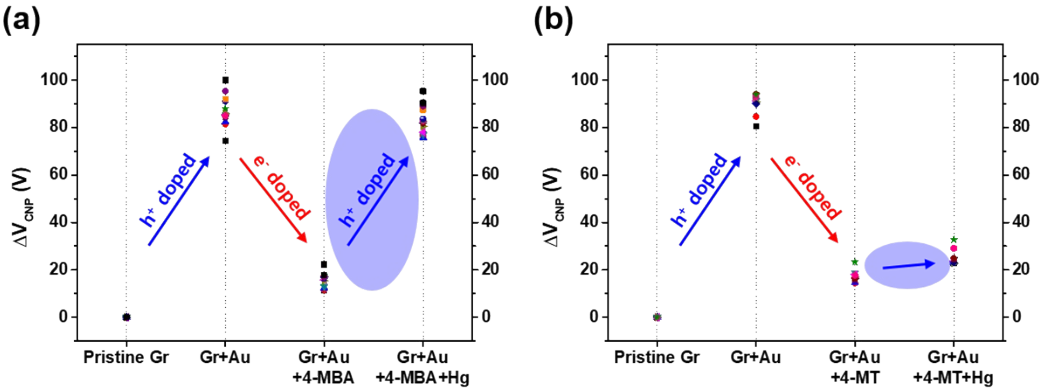

Fig. S5 Charge neutral point value ( ) measured at each step shows that 4-MBA-based FET responds dramatically to the mercury ion treatment (blue shaded circle), while 4-MT (4-mercaptotoluene)-based one responds negligibly. This verifies the strong interaction between carboxyl group and mercury ion.

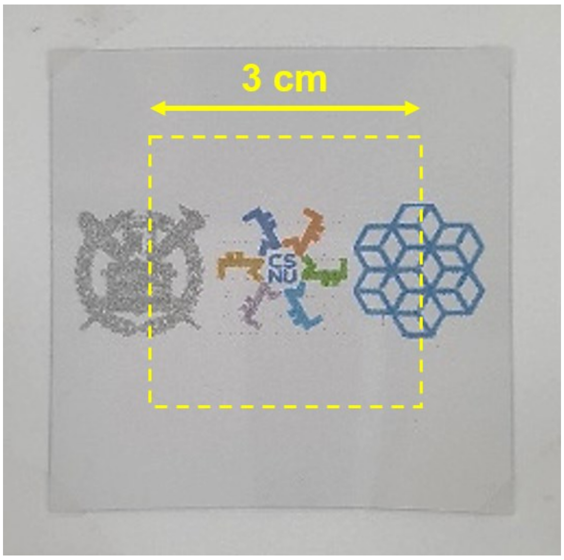

Fig. S6 The pristine graphene transferred on the PET substrate for the measuring sheet resistance. Subsequently, the conditions required by each step of Fig. 1 were dealt with. The sheet resistance of the dashed yellow square area was measured.

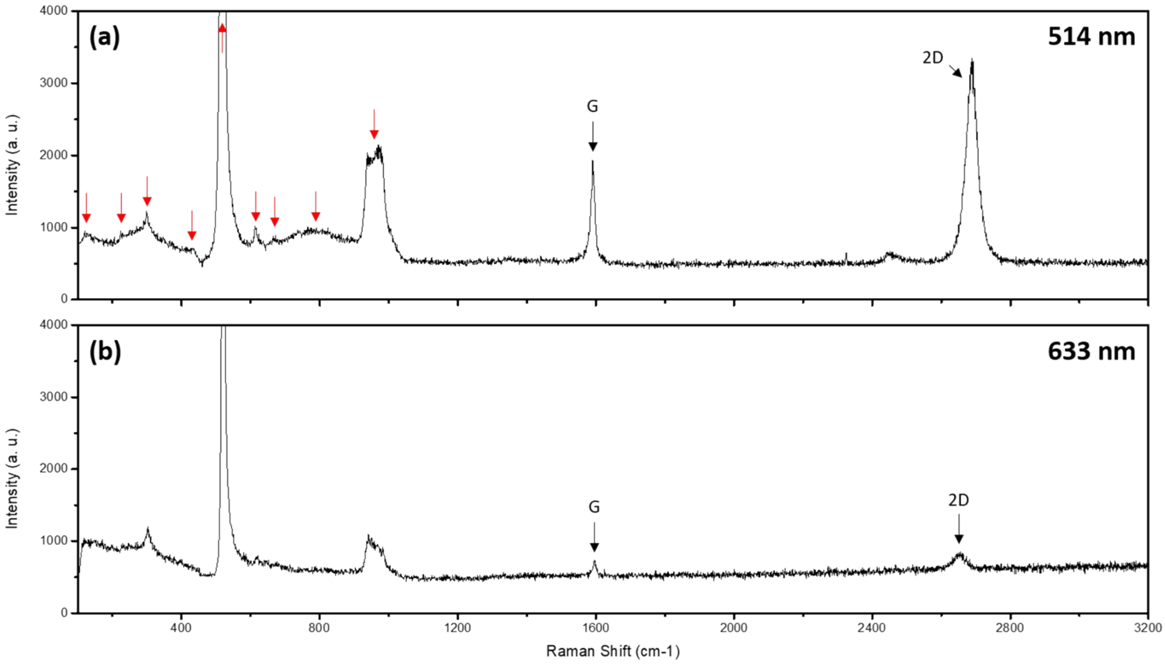

Fig. S7 Raman Spectra of the pristine graphene (step 1 of Fig. 1) by wavelength (a) 514 nm, (b) 633 nm laser. The range of Raman shift in these spectra is full range (100 ~ 3200 cm^{-1}). The positions of the red arrow are the intrinsic peaks of the silicon nanostructure (substrate).

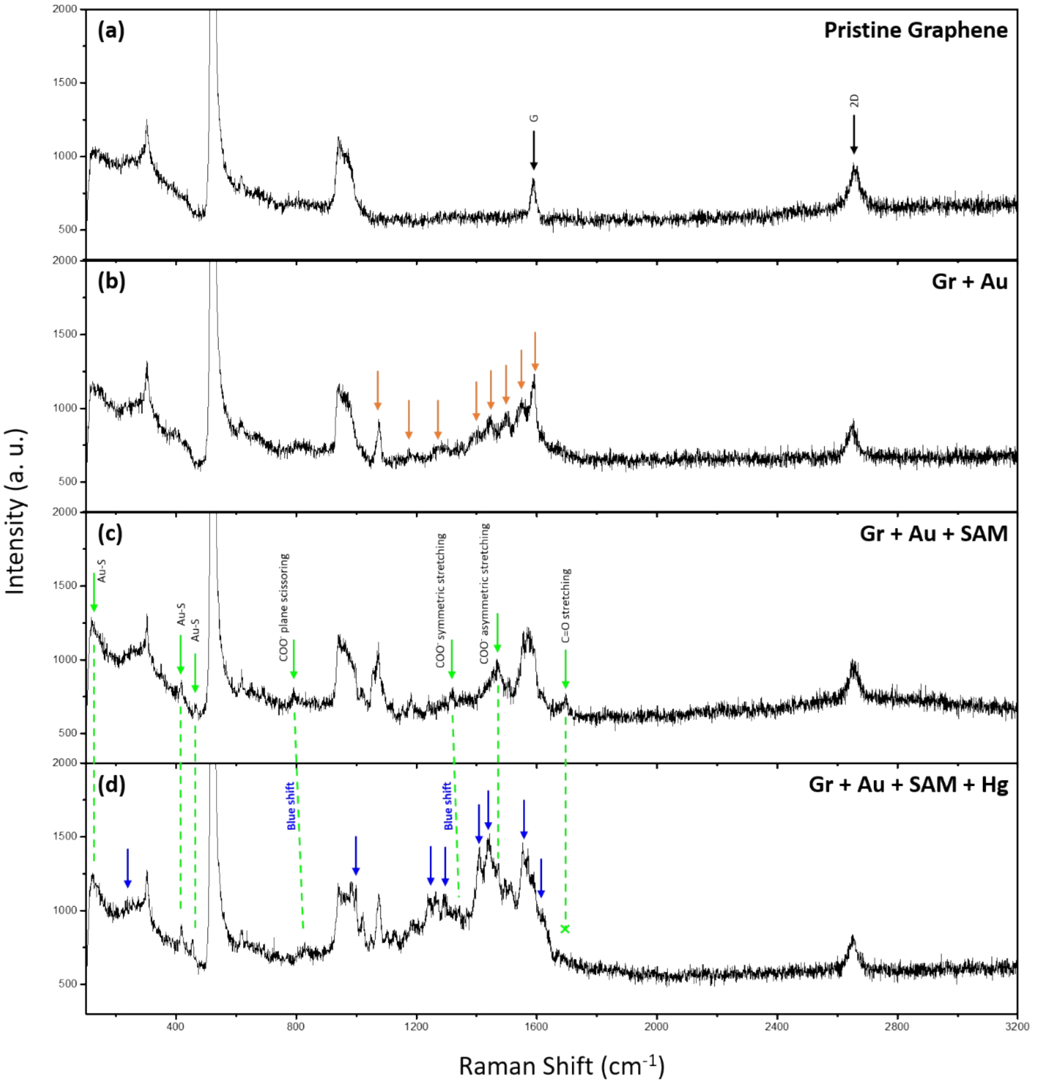

Fig. S8 Raman Spectra of step (a) 1, (b) 2, (c) 3, and (d) 4 of Fig. 1 using 633 nm laser. The range of Raman shift in these spectra is full range (100 ~ 3200 cm^{-1}). The positions of the (b) orange, (c) green, and (d) blue arrow are the peaks by the SERS effect of Au NPs, the Au-S bond or the vibration of carboxyl group of SAM molecules, and SERS effect of Hg^{2+} ions or Hg^{2+} ions chelation by carboxyl group, respectively.

Note S1 Synthesis of graphene

Graphene films were synthesized on a copper foil which has thickness of 25 µm by low pressure CVD method. The carbon precursor is methane gas (50 sccm) and etchant is hydrogen gas (5 sccm). The vacuum level is kept at ~10^{-4} Torr before injecting gases. Using this method, graphene is formed on both sides of the copper foil. To remove a relatively poor-quality graphene on one side, the reactive-ion etching (RIE) was applied on that side: power of 100 W, etching time of 20 s, plasma etchant as oxygen gas (20 sccm) and working pressure of 100 mTorr.

Note S2 Transfer and fabrication of graphene FET devices

The graphene was transferred on substrate through the process of poly (methyl methacrylate) (PMMA) coating, copper etching, transfer on wafer and PMMA removal. First, PMMA layer was spin-coated on the face of copper foil where graphene exists. Then, the substrate was floated on the ammonium persurfate (APS) solution (20 mM with distilled water) in order to etch out copper metal. As a substrate, highly hole-doped Si wafers covered with SiO_2 (300 nm) were prepared. And onto that, the electrodes of FET devices were fabricated by thermal deposition of chrome (3 nm) and gold (50 nm) layers, with using the pre-patterned stencil masks. The PMMA-supported graphene was rinsed with distilled water repeatedly and transferred onto the patterned gold electrodes with care. Then, the substrate was dipped into acetone to remove PMMA for 30 min. Each graphene device was separated by electron beam lithography (EBL), with gate width of 100 μm and gate length of 40 μm. Finally, in order to remove impurities and charge puddles, graphene devices was thermally annealed at 300 ℃ for 3 hours under the atmosphere of hydrogen and argon as etchant (50 sccm) and carrier gas (200 sccm), respectively.

Note S3 Doping of graphene

Hydrogen tetrachloroaurate (III) hydrate (≥99.9%), 4-mercaptobenzoic acid (99%), 4-mercaptophenol (99%), 4-mercaptofluorobenzene (99%), 4-mercaptotoluene (99%), mercury (II) chloride (99.5+%), lead (II) chloride (99.999%), platinum (II) chloride (≥99.9%) and palladium chloride (II) (99%) were purchased from Sigma Aldrich and used without further purification. Gold nanoparticle was deposited on graphene surface by dipping the substrate into the aqueous $HAuCl_4$ solution (0.1 mM or 1 mM, see Fig. S1) for 30 min. Then, the substrates were dipped into ethanolic solution of thiol-SAM molecules (0.1 mM) for 10 min to make SAM on gold nanoparticle surface. The metal ions were captured by the substrate as dipping into the aqueous solutions (0.1 mM) for 10 min at each experiment.

Note S4 Characterization of graphene films

First of all, we proceeded with XPS (AXIS-His, Kratos Analytical, UK) to verify that the graphene made by CVD method was well synthesized, and that the doping was well done by each nanocomponent. The CASA XPS program (CasaXPS 2.3.16 PR 1.6, Casa Software Ltd, UK) was used for the evaluation and analysis of the XPS data using Shirley's method. Electrical property of graphene FET was measured by the 3-terminal (source, drain, and gate) probe station (MS Tech 5500, MS Tech, Korea) at ambient condition. We changed the gate voltage from -40 to +140 V, while keeping the source to drain voltage as 1 mV. (Agilent B2912A, Agilent Technologies, USA). We have tested FET measurements with more than 15 kinds of devices. The Raman spectra measurements were conducted by commercial setup (inVia Raman Microscope, Renishaw, UK) using Ar laser with less than 1 mW of power. The wavelength of laser and grating are 514.5 nm, 2400 gr/mm and 632.8 nm, 1800 gr/mm, respectively. We integrated the signal for 10 s in all measurements for each point. And the Raman spectra mapping (the square parts with a side of 14.5 μm) was completed for about 12 hours. The deposited gold nanoparticles were characterized by a noncontact mode of AFM (XE-100, Park System, Korea). The sheet resistance (mapping) of the monolayer graphene transferred on the PET, in a square with a side of 5 cm, using non-contact method of a sheet resistance measuring device (EddyCus TF map 2525SR, Suragus GmbH, German), and the sheet resistance values inside a center square with a side of 3 cm were extracted (as shown in Fig. 6b). In addition, a 4-point probe portable sheet resistance measuring device (FPP-40k, DASOL ENG, Korea) was used to measure the sheet resistance at a point.
